# Non-Ewald methods: theory and applications to molecular systems

**DOI:** 10.1007/s12551-012-0089-4

**Published:** 2012-09-01

**Authors:** Ikuo Fukuda, Haruki Nakamura

**Affiliations:** 1 RIKEN (The Institute of Physical and Chemical Research), 2-1 Hirosawa, Wako, Saitama 351-0198 Japan; 2 Institute for Protein Research, Osaka University, 3-2 Yamadaoka, Suita, Osaka 565-0871 Japan

**Keywords:** Molecular dynamics, Electrostatic interaction, Reaction field method, Pre-averaging method, Wolf method, Zero-dipole summation method

## Abstract

Several non-Ewald methods for calculating electrostatic interactions have recently been developed, such as the Wolf method, the reaction field method, the pre-averaging method, and the zero-dipole summation method, for molecular dynamics simulations of various physical systems, including biomolecular systems. We review the theories of these approaches and their potential applications to molecular simulations, and discuss their relationships.

## Introduction

Molecular simulation via molecular dynamics (MD) or Monte-Carlo calculations is a powerful tool for understanding the nature of biomolecular systems, including water, proteins, lipids, DNAs, and their complexes. In these simulations, appropriate treatment of the electrostatic interactions is critical, since they play essential roles in a number of systems, by maintaining physical structures, generating chemical properties, and performing biological functions (Patra et al. 2004; Koehl 2006; Reif et al. 2009; Srivastava et al. 2010).

Specifically, the Coulombic electrostatic interaction of *N* atoms with point charges {*q*_1_,…,*q*_*N*_} (the non-SI unit is used, for simplicity) and positions (**r**_1_,…,**r**_*N*_) is

1 $$ E\left( {{{{\bf r}}_1},...,{{{\bf r}}_N}} \right) \equiv \frac{1}{2}\sum\limits_{{i = 1}}^N {\sum\limits_j {\frac{{{q_i}{q_j}}}{{{r_{{ij}}}}}} }, $$

where ${r_{{ij}}} \equiv \left\| {{{{\bf r}}_{{ij}}}} \right\| \equiv \left\| {{{{\bf r}}_i} - {{{\bf r}}_j}} \right\|$ is the distance between atoms *i* and *j*. The manner of summation with respect to *j* depends on the boundary conditions.

Until recently, many of these simulations were performed by using lattice sum (LS) methods such as the Ewald method or its variants, with the assumption of the periodic boundary condition (PBC). At the beginning of these simulation studies, the cutoff truncation method, which is much simpler than the LS method, was frequently used. This is because the monotonic decreasing feature of the Coulombic potential function with increasing *r*_*ij*_ allows the truncation of the interaction (Nicolas et al. 1979; Brooks et al. 1985). However, artifacts of the cutoff method have been pointed out in a number of studies (Patra et al. 2004; Saito 1994). In contrast, it has been mentioned that there are fewer artifacts when applying the PBC used in biophysical system simulations, and thus the PBC would be acceptable for such simulations (de Souza and Ornstein 1997; Buştuğ et al. 2006). Furthermore, a computationally inefficient feature of the LS method has been eliminated, using, e.g., a mesh-based approach. For these reasons, the LS method has been utilized as a standard tool.

However, quite recently, it has been understood that the artifacts of the cutoff method can be sufficiently minimized if a suitable device is added. Such devices take into account specific features, including a system–environment interaction, electrostatic neutrality, and a symmetry of the system, which lead to modifications of the bare Coulomb potential function. Namely, in such a cutoff-based (CB) method, interactions are defined by a certain pairwise function of *r*_*ij*_ within a predetermined cutoff length, and the energy is represented by a finite sum of the pair function and often includes configuration-irrelevant terms. In principle, they are irrelevant to boundary conditions. In contrast, artifacts in applying the PBC to intrinsically non-periodic systems, as well as the problems in the Ewald method and its variants, have been reconsidered. In fact, many biological systems are not intrinsically periodic and have imperfect mathematical periodicity except for certain ideal crystal states.

The CB method is simple and capable, enhancing its straightforward implementation to high-performance computational architectures, including highly parallel protocols and special purpose architectures (Kikugawa et al. 2009). Once the problems of the artifact and the accuracy are solved, the CB method could be more widely used because of its simplicity omitting the long range part of the interactions (Kikugawa et al. 2009; Yonezawa et al. 2011) and the irrelevance to boundary conditions.

A basic criticism of the conventional cutoff method is that an interaction truncation at only about 10–20 Å regarding the slowly decaying Coulombic potential is nothing but a complete artifact. This criticism applies to a system where the particles are spread in a broad area in a vacuum. However, this does not apply in the vivo environment, since many molecules and ions crowd over individual particles. Each positively or negatively charged particle assembles in such a way that the electrostatic interactions cancel each other well, unless very high energy phenomena suddenly occur. This feature should be the same for condensed ionic systems (Clarke et al. 1986). Thus, actual interactions in biological systems are essentially screened, as compared with the bare Coulombic form 1/*r*. In addition, considering the screened nature, we may assume that many biological processes occur through the consecution of adjacent interactions among the particles surrounding every part, rather than through distant, instantaneous interactions. These considerations provide positive motivation for employing the CB methods.

In fact, the CB methods have recently been reconsidered by many researchers, with the use of effective devices (Steinbach and Brooks 1994; Hünenberger and van Gunsteren 1998; Wolf et al. 1999; Yakub and Ronchi 2003; Fukuda et al. 2011). In this review, we discuss these efforts and the relationships among individual methods.

## PBC and the Ewald method

In the 3-dimensional PBCs, the Coulombic energy of *N* charges belonging in an MD cubic unit cell with the cell length *L* is considered to be

2 $$ {E_{\text{PBC}}}\left( {{{{\bf r}}_1},...,{{{\bf r}}_N}} \right) = \frac{1}{2}\sum\limits_{{{{\bf n}} \in {\mathbb{Z}^3}}}^{\prime } {\sum\limits_{{i = 1}}^N {\sum\limits_{{j = 1}}^N } } \frac{{{q_i}{q_j}}}{{\left\| {{{{\bf r}}_{{ij}}} + L{{\bf n}}} \right\|}}. $$

Here, **n** = (*n*_1_,*n*_2_,*n*_3_) is a lattice point represented by three integers, and the prime on the summation indicates the omission of the *i* = *j* term when **n** = (0,0,0). Throughout this review we assume total charge neutrality,

3 $$ \sum\limits_{{i = 1}}^N \,{q_i} = 0, $$

which is critical to defining finite energy. Due to the slow decay of the function, in general, the summation (2) is conditionally convergent, and thus the value is completely dependent on the ordering of the summation. In other words, we should *define* the answer by choosing the ordering. This fact is in total contrast to the absolutely summable summation, where any order gives unique value and thus any (correct) summation method can be used, in principle.

In the periodic system, the Ewald method (Ewald 1921) has been used as the standard. According to de Leeuw et al. (1980), the Ewald energy (except for the dipole term) is interpreted to be a value of the sum, Eq. (2), obtained by a spherical-shell ordering with respect to the copies of the unit cell (i.e., image cells). Namely, the interactions from image cells that are closer to the unit cell in view of the 2-norm [viz., $\left\| {{\bf n}} \right\| \equiv \mathop{{\left\| {{\bf n}} \right\|}}\nolimits_2 \equiv {\left[ {n_1^2 + n_2^2 + n_3^2} \right]^{{1/2}}}$] are counted in a preceding way. Although the counts of the interactions from certain charges that are closer to the unit cell are postponed, the above counting seems to be physically natural (e.g., as compared with counting via the cubic-shell ordering, for which another norm $\mathop{{\left\| {{\bf n}} \right\|}}\nolimits_{\infty } \equiv max\left\{ {\left| {{n_1}} \right|,\left| {{n_2}} \right|,\left| {{n_3}} \right|} \right\}$ is used instead of the 2-norm). This may be the basis for justifying the definition that the Ewald sum is the answer. To effectively handle such an ordering, de Leeuw et al. employed a convergence factor, which adapts well to the ordering, in the original sum, thus yielding a weighted sum in the spherical-shell ordering. In addition, the convergence factor, which contains a parameter, has mathematically good properties that lead to absolute summability and uniform convergence with respect to the parameter.

The Ewald energy traces the properties of the energy, Eq. (2). First, it depends on the cell length *L*: In practice, the Fourier term is influenced so it is smaller for larger values of *L*. While the Ewald energy is invariant under a translation of the axis, this is not the case for a rotation *O*:**r**_*i*_ ↦ O**r**_*i*_ (∀*i*), where the coordinate value of each particle in the unit cell is transformed due to the rotation of the axis. Namely, the energy (of the original system) depends on such a rotation, in general. Invariance is ensured through particular rotations that map ℤ^3^ onto ℤ^3^; e.g. $\tfrac{\pi }{2}$-rotation around the z-axis. The dependences of the energy on the cell-size and the rotation do not necessarily correspond to physical reality. One attempt to recover the rotational invariance is seen in the pre-averaging method, demonstrated in the next section.

The above discussion is focused on possible artifacts generated from the definition of the period. However, once the definition is fixed, the energy upon the period is effectively calculated by the Ewald method (Sagui and Darden 1999). In addition, if the original system permits the periodic structure, then such an artifact can be ignored. However, the application of the PBC to an intrinsically non-periodic system often causes unignorable artifacts.

For an intrinsically non-periodic system such as aqueous protein solutions, the interactions from the infinite copies of the cell, imposed by the PBC, are clearly duplicated (Weber et al. 2000; Kastenholz and Hünenberger 2004), unless we can treat it as a crystal state. As the visible physical effect of such a PBC artifact, enforced stabilization has been observed, e.g., through lower root mean square deviations. Excessively stable alpha-helical structures for explicitly solvated polypeptides (Beck et al. 2005; Lins and Röthlisberger 2006) as well as an entrapment around a non-helical structure in a >20-ns simulation (Lins and Röthlisberger 2006) were found. Erratic phenomena for strain were also observed in nanowires with free surfaces (Gdoutos et al. 2010).

Extensive use of the PBC may be followed by early success in treating an isotropic bulk system. This is because the PBC allows us to avoid the creation of an interface, which often causes significant artifacts, and to mimic the bulk state. However, note that, even in a bulk system, macroscopic fluctuations and wave transport are not necessarily periodic phenomena, given the periods. Although some aspects of these artifacts may be sufficiently reduced by careful consideration of the simulation conditions, such as the cell size, dielectric constant, charge distribution, and the sampling duration, the issues are still under discussion (Hünenberger and McCammon 1999; Monticelli et al. 2006).

Finally, we note there are alternative approaches such as considering the Poisson equation in the PBC. Several methods, including those to attain a fast convergence, have been intensively developed (Tyagi 2005; see also the references therein).

## CB methods

We first discuss general issues, including a truncation mode, function smoothing technique, and artifacts we should consider in cutoff approaches. Second, as a specific issue, individual CB methods are discussed, mainly for recently developed ones. Here, we address the reaction field (RF) method, the pre-averaging (PA) method, the Wolf method, and the zero-dipole (ZD) summation method.

To specifically perform a cutoff, as well as the pair potential function itself, a cutoff truncation mode (i.e. how to truncate the interactions) should be fixed. This is not trivial, because the choice affects the simulation results and often causes significant artifacts. In the atom-based cutoff (AC) mode, for atom *i*, the contribution from atom *j* such that *r*_*ij*_ > *r*_*c*_ is simply ignored. In the group-based cutoff (GC) mode, all atom–atom interactions between any two molecules should be on or off, according to a certain “marker” being inside or outside the cutoff sphere, respectively. Such a marker is usually chosen to be a certain center of the molecule (Baumketner 2009; Chipot et al. 1994; Neumann 1985; Schreiber and Steinhauser 1992a; van der Spoel et al. 1998), a certain atom (Alper and Levy 1989; Leach 2001), or a certain distance (Fukunishi et al. 2003; Leach 2001). Some implementations consider the marker out to a distance several Angstroms beyond the cutoff distance (this is often combined with a pair-list recycling feature). Smoothed on–off is also possible (Steinbach and Brooks 1994), and a certain-defined (e.g., neutrally charged) atom group is usually used (Wohlert and Edholm 2004), instead of a whole molecule.

Biophysical system needs an aqueous environment. Although cutoff methods have been applied to water(-like) systems, the results of the dielectric property, which would be the most sensitive property to a treatment of the electrostatic interaction (Andrea et al. 1983), often involve significant artifacts. This is particularly for the distance-dependent Kirkwood factor *G*_K_(*r*), which is the ensemble average of the dipole–dipole angle distribution in the sphere of radius *r* (Steinhauser 1982; Höchtl et al. 1998). Many cutoff methods (Yonetani 2006) yielded *G*_K_(*r*) with a significant hole-like structure around *r*_c_, contradicting the expected results (Mark and Nilsson 2002).

Such artifacts appear for several reasons. First, earlier simulations suffered from limited computational time. That is, the dielectric properties require a long simulation duration, typically over a ns, to yield reliable results, because of the slow convergence of the (time) ensemble average (Heinz et al. 2001; Li et al. 2007; Gereben and Pusztai 2011). Second, ad hoc procedures, such as velocity rescaling to stabilize the system, are not recommended for evaluating sensitive properties. Finally, and often critically, the discrepancies are prominent in the GC mode, rather than the AC mode. In fact, the artifacts intrinsic in many GC modes have been pointed out. The energetic and statistical jumps were discussed (Steinhauser 1982). A clear explanation by Baumketner (2009) mentions the generation of an artificial dipole layer on the cutoff sphere of each atom: the interaction unbalances by the GC mode disrupt the charge compensation near the cutoff surface (Hummer et al. 1997).

However, as many molecular simulations utilize the GC mode in cutoff methods, there are several reasons to use it: (1) a force-field is usually developed on interactions between molecules; (2) it can prevent large energy fluctuations near *r*_c_, which are encountered in the straight AC, because individual inter-atom interactions are often significantly large (Steinbach and Brooks 1994; Leach 2001); (3) if both molecules are neutral, then the leading term of their interaction can be described by a dipole–dipole interaction [∼(*r*_MM_)^−3^], whose enhanced screening feature conforms to justify the cutoff (Wohlert and Edholm 2004); and (4) it is preferable for the RF method (see below) to attain the assumption of charge neutrality in the cavity, when every molecule is neutral (Neumann 1985).

If these issues can be addressed using the AC mode with certain other devices, then we expect the AC mode will be used to avoid the artifacts in the GC mode. Potential (or force) smoothing techniques (see, e.g., Steinbach and Brooks 1994) are useful to address issues (1) and (2). In fact, the artifacts in the dielectric properties of a water system using the GC mode were reduced by using the AC mode when employing smoothing methods, involving the force-switching or the force-shifting (Mark and Nilsson 2002; van der Spoel and van Maaren 2006). In the other systems, an artifact in explicitly solvated peptide conformation in the GC mode cutoff was obtained (Schreiber and Steinhauser 1992a), but physically reasonable behavior was confirmed in the AC mode using a force-shifting method (Beck et al. 2005). An oligonucleotide in aqueous solution can be stabilized via the AC mode with smoothing methods such as the force shift, although it failed in the GC mode (Norberg and Nilsson 2000). The issues (3) and (4) will be considered in specific CB methods.

## The reaction field method

During the long history of the RF method (Onsager 1936), its effectiveness and artifacts have both been pointed out (Barker and Watts 1973; Steinhauser 1982; Neumann 1983; Hünenberger and van Gunsteren 1998; Essex 1998; Hansson et al. 2002; Gargallo et al. 2003; Robertson et al. 2008; Schulz et al. 2009; Míguez et al. 2010). The method can be viewed as a modification of the simple truncation method. It takes into account the interactions between each atom (or molecule) and the environment outside its cutoff sphere. Specifically, we consider a “cavity” of each molecule *a* (the “cavity” resembles a cutoff sphere with radius *r*_c_ for each atom; see assumption (ii) below), with the region outside the cavity assumed to be a dielectric continuum, with dielectric constant *ε*_RF_, polarized by reacting with the molecules inside the cavity. The polarization generates an electric field (reaction field) *E*_*a*_ represented by

4 $$ {E_a} \equiv \frac{{2({\varepsilon_{\text{RF}}} - 1)}}{{2{\varepsilon_{\text{RF}}} + 1}}\frac{1}{{r_{\text{c}}^3}}\sum\limits_b {{\mu_b}}, $$

where the summation is over all molecules in the cavity of molecule *a* and ${\mu_b} \equiv \sum\nolimits_{{i \in {\text{Mo}}{{\text{l}}_b}}} \,{q_i}{{{\bf r}}_i}$ is the dipole of molecule *b* (Fröhlich 1958; Allen and Tildesley 1987). Assume that (i) the total charge in each cavity equals zero, and (ii) each molecule *a* is sufficiently small such that the set of atoms in the cavity of molecule *a* equals the atoms in the cutoff sphere of atom *i*, for *every i* of molecule *a*. The RF energy of the system of *M* molecules is thus shown to be

5 $$ {E^{\text{RF}}}\left( {{{{\bf r}}_1},...,{{{\bf r}}_N}} \right) \equiv - \frac{1}{2}\sum\limits_{{a = 1}}^M {\left( {{\mu_a}|{E_a}} \right)} = \frac{1}{2}\sum\limits_{{i = 1}}^N {\sum\limits_{\matrix{\scriptstyle j( \ne i) \\ {r_{{ij}}} < {r_{\text{c}}} }<!endsubarray> } {{q_i}{q_j}\frac{{{\varepsilon_{\text{RF}}} - 1}}{{2{\varepsilon_{\text{RF}}} + 1}}\frac{{r_{{ij}}^2}}{{r_{\text{c}}^3}}} } . $$

Hence, the total energy is

6 $$ {E_{\text{RF}}}\left( {{{{\bf r}}_1},...,{{{\bf r}}_N}} \right) \equiv \frac{1}{2}\sum\limits_{{i = 1}}^N {\sum\limits_{\matrix{\scriptstyle j( \ne i) \\ {r_{{ij}}} < {r_{\text{c}}} }<!endsubarray> } {\frac{{{q_i}{q_j}}}{{{r_{{ij}}}}}} } + {E^{\text{RF}}}\left( {{{{\bf r}}_1},...,{{{\bf r}}_N}} \right) = \frac{1}{2}\sum\limits_{{i = 1}}^N {\sum\limits_{\matrix{\scriptstyle j( \ne i) \\ {r_{{ij}}} < {r_{\text{c}}} }<!endsubarray> } {{q_i}{q_j}{V_{\text{RF}}}({r_{{ij}}}),} } $$

with

7 $$ {V_{\text{RF}}}(r) \equiv \frac{1}{r}\left\{ {1 + \frac{{{\varepsilon_{\text{RF}}} - 1}}{{2{\varepsilon_{\text{RF}}} + 1}}\mathop{{\left( {\frac{r}{{{r_{\text{c}}}}}} \right)}}\nolimits^3 } \right\}. $$

Considering its application to MD, however, the non-smoothness of the energy causes problems, such as the energy non-conservation in *NVE* simulations. Although the physical basis is rather unclear, the potential-shifted method may serve as a remedy; viz.,

8 $$ {V_{\text{RF}}}(r) - {V_{\text{RF}}}({r_{\text{c}}}) $$

is used instead of *V*_RF_(*r*) for *r* < *r*_c_, and 0 is set for *r* ≥ *r*_c_. The continuity of the potential function at *r* = *r*_c_ is thus gained, but the force function, defined by *F*_RF_(*r*) ≡ −*DV*_RF_(*r*) for *r* < *r*_c_ and *F*_RF_(*r*) ≡ 0 for *r* ≥ *r*_c_, does not share in the benefit. In fact, *F*_RF_ is continuous at *r*_c_ only in the limit of *ε*_RF_ → ∞. Of course, we can treat Eq. (5) as a molecule–molecule interaction, instead of an atom–atom (site–site) interaction. In any case, the energetic non-smoothness is a significant problem in the MD simulation. In fact, the atomic velocities were rescaled at every certain timing, to prevent energy or temperature drift (Neumann 1985; Alper and Levy 1989). Although several smoothing procedures are available, an appropriate correction to the total energy, to address the potential-energy deformation, should generally be made.

Here, we discuss the remaining issues addressed in the GC mode requirements. As for issue (4), note that combination of the GC mode with the neutrality of each molecule is a sufficient, but not necessary, condition for assumption (i). Furthermore, other theoretical derivations or interpretations may not need such assumptions. In fact, the derivation of RF energy can be achieved without reference to the dipolar interactions (Tironi et al. 1995). Several methods, utilizing such as the Poisson equation, and extensions have been demonstrated (Hünenberger and van Gunsteren 1998; Perram and Smith 1987; Nakamura 1996). In addition, in the ZD summation method, described later, the RF method in the case of *ε*_RF_ → ∞, which is the most suitable case in view of the force-function continuity as stated, naturally arises, and issues (3) and (4) are addressed with the AC mode.

**Fig. 1:**
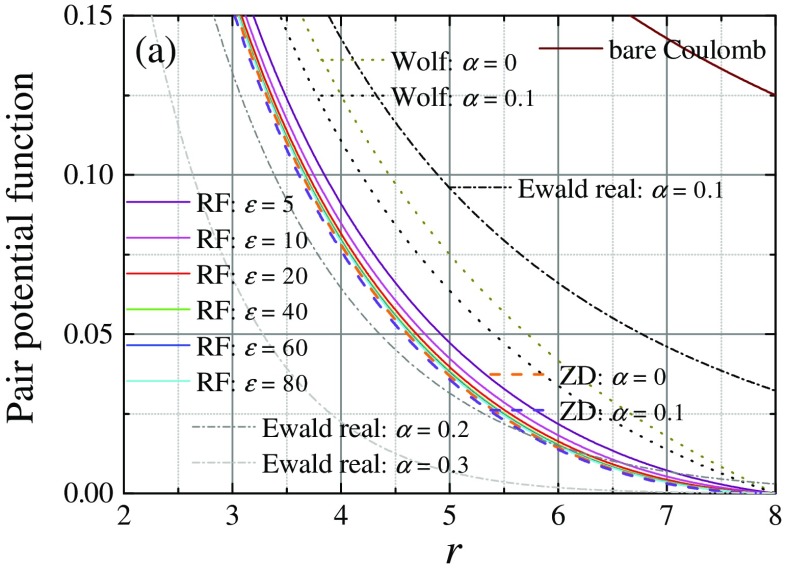
Pair potential functions for CB methods with the cutoff length of *r*_c_ = 8. For the RF method, Eq. (8) is shown with the dielectric parameter *ε* ≡ *ε*_RF_ (curves over *ε* = 40 are indistinguishable). Functions are shown for the Wolf method, *V*(*r*) − *V*(*r*_c_) in Eq. (11); the ZD summation method, *u*(*r*) − *u*(*r*_c_) in Eq. (15). For comparison, Eq. (12), the real part of the Ewald method, and the bare Coulomb, 1/*r*, are shown

The RF method has also been applied to aqueous biosystems, and some of the properties were well reproduced. However, it still often generates significant artifact in the distance-dependent Kirkwood factor (Neumann 1985; Belhadj et al. 1991). As stated, also in the RF method, the artifacts in the GC mode (Neumann 1986; Alper and Levy 1989) were reduced by using the AC mode (Hünenberger and van Gunsteren 1998; Schulz et al. 2009). To reduce artifacts in the RF method, a large dielectric constant *ε*_RF_ may be used. In fact, the deviated structure in *G*_K_(*r*) in water system is reduced as *ε*_RF_ increases (van der Spoel and van Maaren 2006). Schulz et al. (2009) obtained good results in the case of *ε*_RF_ ≡ ∞. The infinite value has also been used (Neumann 1986; Essex 1998; Míguez et al. 2010; Schreiber and Steinhauser 1992b). For the water system, the potential curves with *ε*_RF_ ∼ 80 and *ε*_RF_ ≡ ∞ a re similar (Fig. 1), and so the approximation of *ε*_RF_ ≡ ∞ may be more suitable. Note that the very low dielectric constant (around 3) for proteins is derived from dried, powdered protein data. The effective value for real solvated proteins is much closer to that of water (Beck et al. 2005), and is 10 to 50, depending on the pair separation distance.

There may be several other limitations in the RF method. The physically unnatural assumption of the instantaneous response by the RF in its derivation has been discussed (van Gunsteren et al. 1978). Homogeneity of the system may be required to represent the environment of each particle as a constant dielectric. However, the RF method was shown to be effective in evaluating an inhomogeneous system. For example, the interfacial properties of different water models were well described (Míguez et al. 2010), and a stable simulation using the AC mode was conducted in a solvated DNA system (while the GC mode failed; Ni and Baumketner 2011).

## The pre-averaging method

The pre-averaging procedure was introduced by Yakub and Ronchi (2003), to remove the artificial cubic symmetry in the LS method and recover the rotational invariance. Their energy formula was obtained using the Ewald summation expansion and by averaging the quantities in the expansion over spherical angular coordinates. This corresponds to a situation treating a uniformly distributed system. The energy formula is

9 $$ {E_{\text{PA}}}\left( {{{{\bf r}}_1},...,{{{\bf r}}_N}} \right) \equiv \frac{1}{2}\sum\limits_{{i = 1}}^N {\sum\limits_{\matrix{\scriptstyle j( \ne i) \\ {r_{{ij}}} < {r_{\text{m}}} }<!endsubarray> } {{q_i}{q_j}\frac{1}{{{r_{{ij}}}}}\left\{ {1 + \frac{1}{2}\frac{{{r_{{ij}}}}}{{{r_{\text{m}}}}}\left[ {\mathop{{\left( {\frac{{{r_{{ij}}}}}{{{r_{\text{m}}}}}} \right)}}\nolimits^2 - 3} \right]} \right\}} } - \frac{3}{{4{r_{\text{m}}}}}\sum\limits_{{i = 1}}^N {q_i^2,} $$

where *r*_m_ is the radius of the volume-equivalent sphere of the MD cubic cell with edge *L*:

10 $$ \frac{4}{3}\pi r_{\text{m}}^3 = {L^3}. $$

The PA method has been very successfully applied (Yakub et al. 2007; Arima et al. 2009; Jha et al. 2010). It yields accurate energy in disordered systems, including a one-component plasma and a two-component fluid (Yakub and Ronchi 2005; Yakub 2006), and, further, in non-spherical, crystal systems (Yakub and Ronchi 2003; 2005). Moreover, Guerrero-García et al. (2011) applied the PA method to inhomogeneous systems, constituted by two fixed nanoparticles immersed in a size-asymmetric monovalent electrolyte.

However, the cutoff length, *r*_m_, intrinsic to the PA formalism, causes severe practical limitations in certain cases. Namely, since *r*_m_ is proportional to the cell size *L* and is larger than *L*/2, enormous computational efforts will be required to treat a large system, for example in biological systems where the size is >nm. Reinterpretation of the cutoff length is possible in the ZD summation method, as described below. See also the recent work by Vernizzi et al. (2011).

## The Wolf method

Here, we briefly review the Wolf method and its variants, for which the effectiveness in terms of the accuracy and computational cost have been demonstrated in many applications (Wolf et al. 1999; Demontis et al. 2001; Zahn et al. 2002; Fennell and Gezelter 2006; Avendaño and Gil-Villegas 2006; Sepliarsky et al. 2006; Ribeiro 2007; Desai 2007; Goto et al. 2007; Mahadevan and Garofalini 2007; Nagata and Mukamel 2010; Chen et al. 2010; Kuang and Gezelter 2010; Gdoutos et al. 2010; Chevrot et al. 2011; Kannam et al. 2012; Méndez and Villegas 2012).

In seeking the Madelung energies of crystal systems via the straight cutoff method, Wolf (1992) and Wolf et al. (1999) showed that the energy exhibited very slow convergence and oscillated around the exact value as the cutoff length increased, but the value was very accurate only when certain cutoff lengths, characteristic of individual crystal structures, were adopted. They found that this feature was not only due to the oscillating feature but also to the (near) achievement of the charge neutrality in the cutoff spheres with those characteristic cutoff lengths. They also found that the energy error was nearly proportional to the net charge in each sphere. These observations led to the idea of the subtracting the interactions by excess charges in the cutoff sphere, ${q_i}\left( {\sum\nolimits_{{j,\;{r_{{ij}}} < {r_{\text{c}}}}} \,{q_j}} \right)/{r_{\text{c}}}$, from the straight pairwise sum. Here, for the excess charges, their total quantities are equal to the net charge in the sphere, and their positions are assumed to be at the surface. Hence, they led to a formula for the electrostatic energy,

11 $$ {E_{\text{Wolf}}}\left( {{{{\bf r}}_1},...,{{{\bf r}}_N}} \right) \equiv \frac{1}{2}\sum\limits_{{i = 1}}^N {\sum\limits_{\matrix{\scriptstyle j( \ne i) \\ {r_{{ij}}} < {r_{\text{c}}} }<!endsubarray> } {{q_i}{q_j}\left[ {V({r_{{ij}}}) - V({r_{\text{c}}})} \right]} } - \left[ {\frac{{V\left( {{r_{\text{c}}}} \right)}}{2} + \frac{\alpha }{{\sqrt {\pi } }}} \right]\sum\limits_{{i = 1}}^N {q_i^2.} $$

Here, instead of 1/*r*, the damped function,

12 $$ V(r) \equiv \frac{{{\text{erfc}}(\alpha r)}}{r}, $$

was introduced, since it was very effective to achieve a fast convergence. The last term was derived by an approximation, $\tfrac{1}{2}\sum\nolimits_{{i = 1}}^N {\sum\nolimits_{{j( \ne i)}} {{q_i}{q_j}\left[ {1/{r_{{ij}}} - V\left( {{r_{{ij}}}} \right)} \right]} }$ $\sim - (\alpha /\sqrt {\pi } )\sum\nolimits_{{i = 1}}^N \,q_i^2$, for a small damping factor *α*(≥ 0) (Wolf et al. 1999; but see Angoshtari and Yavari 2011).

Equation (11) can be viewed as considering the contributions from image charges; viz., for every atom in the cutoff sphere around *i*, the opposite signed image-charge exists on the surface and interacts with (only) *i*. Since the straightforward differentiation of Eq. (11) leads to the discontinuous scheme at *r* = *r*_c_, further consideration is needed to define an atomic force for MD. Analogous to the effective potential *V*(*r*) − *V*(*r*_c_) reflecting the view of the image charges, one might suppose an effective force function, ${F_{\text{Wolf}}}(r) \equiv - \left[ {DV(r) - DV\left( {{r_{\text{c}}}} \right)} \right]$. Although this “force” satisfies the continuity, unfortunately it is not compatible with the energy function Eq. (11). These problems arise from a straightforward interpretation of the image-charge picture.

The second view of Eq. (11) can be obtained when one considers its first term to be the shifted-potential method for *V*. In developing such a potential-deformation picture (Zahn et al. 2002; Fennell and Gezelter 2006), the shifted-force method for the force − *DV* was proposed. This means that the force function is given by *F*_Wolf_, and that the corresponding potential energy is constructed by the integration. Many physical properties can be traced in these approaches. However, note that the physical basis would be unclear, and the treatment of the self image term (2nd term in Eq. (11)) becomes ambiguous.

The third view of Eq. (11) is that the energy can be obtained from a suitably-defined neutralized summation. That is, it is derived from the assertion that the interaction contribution should be counted in a *neutralized subset L* ^*i*^ (including *i*), whose existence is assumed and characterized where: (a) any particle in *L* ^*i*^ is inside the cutoff sphere; (b) the total charge in *L* ^*i*^ is zero; and (c) a particle not belonging to *L* ^*i*^ but inside the sphere is located close to the cutoff surface. Based on this physically clear view, the force-switching Wolf method (Fukuda et al. 2008) established an approximation to the neutralized summations, and the energy becomes

13 $$ {E_{{{\text{FSw}} - {\text{Wolf}}}}}\left( {{{{\bf r}}_1},...,{{{\bf r}}_N}} \right) \equiv \frac{1}{2}\sum\limits_{{i = 1}}^N {\sum\limits_{\matrix{\scriptstyle j( \ne i) \\ {r_{{ij}}} < {r_{\text{c}}} }<!endsubarray> } {{q_i}{q_j}[\widehat{V}({r_{{ij}}}) - \widehat{V}({r_{\text{c}}})]} } - \left[ {\frac{{\widehat{V}\left( {{r_{\text{c}}}} \right)}}{2} + \frac{\alpha }{{\sqrt {\pi } }}} \right]\sum\limits_{{i = 1}}^N {q_i^2,} $$

where

14 $$ \widehat{V}(r) \equiv \left\{ {\matrix{ {V(r)} &{\text{for}} &{0 < r < {r_1}} \\ {{V^{ * }}(r) + V\left( {{r_1}} \right) - {V^{ * }}\left( {{r_1}} \right)} &{\text{for}} &{{r_1} \leqslant r \leqslant {r_{\text{c}}}} \\ {V\left( {{r_1}} \right) - {V^{ * }}\left( {{r_1}} \right)} &{\text{for}} &{{r_{\text{c}}} < r < \infty } \\ }<!end array> } \right., $$

with *r*_1_ being the switching length and *V* ^*^ a suitable switching function. This provides consistent potential and force functions, and smoothness for safely conducting MD simulations. This is simply the Wolf formula [Eq. (11)] with $\widehat{V}$ instead of *V* (Eq. (11) is recovered as *r*_1_ → *r*_c_). It was applied to calculate the free energies of an alanine dipeptide in explicit water, and reliable results were obtained (Yonezawa et al. 2011).

## The ZD summation method

In developing the neutralizing principle, the ZD summation method (Fukuda et al. 2011) provides the energy derived by counting the interactions for a neutralized subset regarding the dipoles as well as the charges. Thus, the ZD summation method can be viewed as an extension of the Wolf method. It effectively avoids the nonzero-dipole and nonzero-charge state artificially generated in the simple cutoff scheme. Its physical basis is clear, and the axiomatic approach ensures there is no confusion in defining its energy,

15 $$ {E_{\text{ZD}}}\left( {{{{\bf r}}_1},...,{{{\bf r}}_N}} \right) = \frac{1}{2}\sum\limits_{{i = 1}}^N {\sum\limits_{\matrix{\scriptstyle j( \ne i) \\ {r_{{ij}}} < {r_{\text{c}}} }<!endsubarray> } {{q_i}{q_j}\left[ {u\left( {{r_{{ij}}}} \right) - u\left( {{r_{\text{c}}}} \right)} \right]} } - \left[ {\frac{{u\left( {{r_{\text{c}}}} \right)}}{2} + \frac{\alpha }{{\sqrt {\pi } }}} \right]\sum\limits_{{i = 1}}^N {q_i^2,} $$

where

16 $$ u(r) \equiv V(r) - \frac{1}{2}\frac{{DV\left( {{r_{\text{c}}}} \right)}}{{{r_{\text{c}}}}}{r^2}. $$

Equation (15) is simple enough, and also takes the form of Eq. (11) using *u* instead of *V*. In addition to the information obtained in the zero charge scheme by the Wolf approach such that the excess charge is on the cutoff sphere, the zero dipole condition adds information about the states, which helps to improve the accuracy. Furthermore, this scheme accepts the AC mode well, and the above-mentioned four issues regarding the GC mode are sufficiently addressed. In fact, the pair function smoothly tends to zero at *r*_c_, and the remaining issues (3) and (4) can be cleared by including the damping factor and considering the neutralized condition through the whole cutoff sphere, rather than each molecule.

### Relationship to other CB methods

Thus far, we have discussed the RF, PA, and Wolf methods. These methods differ in their concepts, derivations, and the energy formulae themselves. Surprisingly, we see that they are related to each other by considering the connection of the individuals to the ZD summation method, through special limits of the parameters.

As well as the fact that the ZD summation method is an ideaistic extension of the Wolf method, function *u* [Eq. (16)] tends to function *V* [Eq. (12)] as *r*_c_ → ∞, and the difference of the pair potentials and that of the constant terms between the two methods approach 0 as *r*_c_ → ∞ (they also approach 0 as *α* → ∞).

For the PA method, note that its energy, Eq. (9), can be rewritten as

17 $$ {E_{\text{PA}}}\left( {{{{\bf r}}_1},...,{{{\bf r}}_N}} \right) = \frac{1}{2}\sum\limits_{{i = 1}}^N {\sum\limits_{\matrix{\scriptstyle j( \ne i) \\ {r_{{ij}}} < {r_{\text{c}}} }<!endsubarray> } {{q_i}{q_j}\left[ {{V_{\text{PA}}}\left( {{r_{{ij}}}} \right) - {V_{\text{PA}}}\left( {{r_{\text{m}}}} \right)} \right]} } - \frac{{{V_{\text{PA}}}\left( {{r_{\text{m}}}} \right)}}{2}\sum\limits_{{i = 1}}^N {q_i^2,} $$

where ${V_{\text{PA}}}(r) \equiv \left( {1/r} \right)\left\{ {1 + \left( {\tfrac{1}{2}} \right){{\left( {r/{r_{\text{m}}}} \right)}^3}} \right\}$. We see that the energy of the ZD summation, Eq. (15), gives Eq. (17) when we set *α* = 0 and *r*_c_ = *r*_m_, noting that the potential function *u* then corresponds to *V*_PA_. This fact enhances the free use of the cutoff length, which is not limited to *r*_m_, in the PA method, and might explain why the PA method yields “surprisingly positive” results in anisotropic systems (Yakub and Ronchi 2003).

In the RF method, a pairwise sum is conducted by *V*_RF_ [Eq. (7)] (Neumann 1985) or by the shifted form *V*_RF_ − *V*_RF_(*r*_c_) [Eq. (8)] (van der Spoel and van Maaren 2006; Baumketner 2009). Note that *V*_RF_ with *ε*_RF_ → ∞ corresponds to *u* with *α* = 0. In other words, if we propose a modified RF summation by

18 $$ {E_{\text{MRF}}}\left( {{{{\bf r}}_1},...,{{{\bf r}}_N}} \right) \equiv \frac{1}{2}\sum\limits_{{i = 1}}^N {\sum\limits_{\matrix{\scriptstyle j( \ne i) \\ {r_{{ij}}} < {r_{\text{c}}} }<!endsubarray> } {{q_i}{q_j}\left[ {{V_{\text{RF}}}\left( {{r_{{ij}}}} \right) - {V_{\text{RF}}}\left( {{r_{\text{c}}}} \right)} \right]} } - \frac{{{V_{\text{RF}}}\left( {{r_{\text{c}}}} \right)}}{2}\sum\limits_{{i = 1}}^N {q_i^2,} $$

then its limit as *ε*_RF_ → ∞ equals the ZD summation energy (15) with *α* = 0. The difference between the RF energy and the modified one is the “self energy,” viz. the last term in Eq. (18).

## Other methods

Other promising CB approaches exist. The ZD summation method can be extended to accommodate higher multipoles. The force-matching method (Ercolessi and Adams 1994; Shi et al. 2008), the isotropic periodic summation (Wu and Brooks 2005; 2008), and the screening scheme using the Yukawa potential (Carré et al. 2007) are highly effective. Important new non-Ewald electrostatics methods have been developed, including local molecular field theory (Chen and Weeks 2006), a fast multipole method combined with a reaction field (Mathias et al. 2003), the lattice-sum-emulated reaction-field method (Heinz and Hünenberger 2005), an image-charge reaction field method (Lin et al. 2009, 2011), and a model of electrostatic and liquid-structure forces (Hassan 2007).

## Conclusion

After reviewing intrinsic aspects of the PBC with the LS method, we mainly considered the CB methods including the conventional RF method and the recently-developed PA, Wolf, and ZD summation methods.

Although the PBC with the LS method has been most frequently applied to biomolecular systems, they are still far from reality, and some artifacts have been recognized. The CB method could provide a promising solution, and, because of its simple features, it could satisfy the demands of high-performance computational architectures. Investigation of the RF method using the AC mode should be continued. Pursuit of symmetry of the system, as considered in the PA method, will be useful in particular for reconsidering the boundary conditions. Although more considerations may be required, the relationships between the CB methods suggest new interpretations and extensive applications. The “interactions” among the individual CB methods, even with the LS method, should mature the algorithm for calculating electrostatic interactions.
